# Malignant Schwannoma in patients with von Recklinghausen disease: report of two cases

**DOI:** 10.1016/S1808-8694(15)30851-X

**Published:** 2015-10-18

**Authors:** Vinicius Antunes Freitas, Michel Cyrino Saliba, Eduardo Cesar Dolabela de Moraes, Gabriela Amélia Nassif de Morais Teixeira, João Batista de Oliveira

**Affiliations:** 1 MD. ENT Resident - Núcleo De Otorrino BH.; 2 MD. ENT Resident - Núcleo De Otorrino BH.; 3 MD. ENT Resident - Núcleo De Otorrino BH.; 4 MD. Otorhinolaryngologist.; 5 MSc. Otorhinolaryngologist and Head and Neck Surgeon, ENT preceptor - Santa Casa de BH and Núcleo de Otorrino BH. Núcleo de Otorrino BH.

**Keywords:** neurilemmoma

## INTRODUCTION

Von Recklinghausen’s disease is characterized by the presence of cafe-au-lait spots, multiple neurofibromas and hamartomas. It is a dominant autosomal disease with high penetrance and variable expression, involving the head and neck in 22 to 47% of the patients. It is known as type I neurofibromatosis, in order to differentiate it from central involvement, especially that of the eight cranial nerve (acoustic neuroma - type 2 neurofibromatosis)^1^, ^2^.

Malignant transformation varies between 2% and 40%, and these patients are more prone to having malignant tumors of the nervous tissues and other secondary neoplasias^1^. They tend to appear in young patients in the middle of their bodies, and these patients run a high risk of having the tumors turn malignant - of worse prognoses and happening earlier on when compared to neurofibromas alone^1^, ^3^, ^4^.

Malignancy must be suspected when there is a fast growing and painful mass, investigated by image exams (CT, MRI, Bone Scintigraphy and Angiography) for staging, resection evaluation and lesion biopsy purposes^3^. Benign lesions can also show fast growth^3^, ^5^.

Clinical development is characterized by local recurrences, and has poor prognosis in patients with multiple neurofibromatosis, and the lungs are frequently the seat of distant metastasis^4^.

Treatment is based on radiotherapy, chemotherapy and surgery; it bears a low 3-year survival rate^4^.

## CASE REPORTS

### Case 1

**Figure 1:**
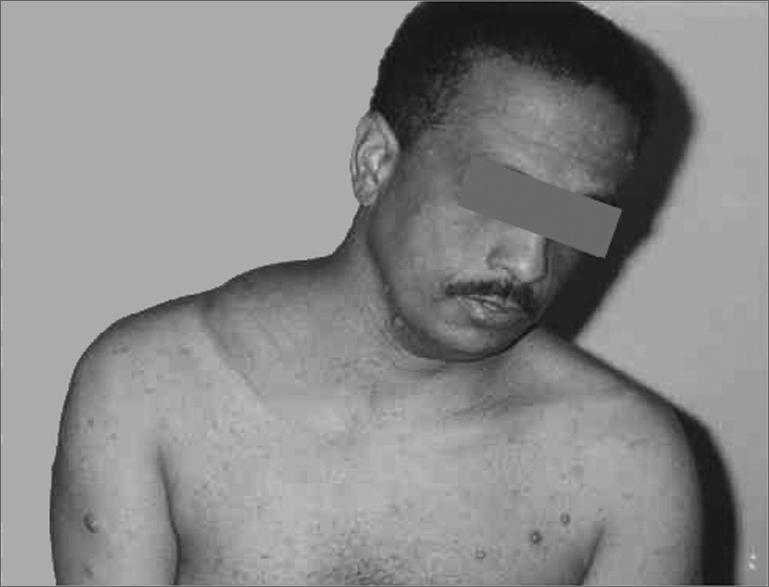
Neck Schwannoma

C.E.R., 37 years, single, Caucasian, with previous diagnosis of von Recklinghausen’s disease and a history of a fast-growing mass in the right supraclavicular fossa for one year (Fig. 1). Physical exam: 15 cm nodular, hard, apparently well outlined, painful at palpation lesion, not adhered to the deep planes. The patient was submitted to surgery, and a tumor was found adhered to the clavicle periosteum and to the brachial plexus. We carried out a radical neck lymph node resection. Macroscopic aspect: nodular lesion, of 9x8x7 cm, cross-sections with cystic and solid white-yellowish soft areas.

Microscopy: malignant mesenchymal tumor made up of ovoid and spindle-like core cells, coated by an elongated cytoplasm, creating multidirectional bundles, with a moderate mitotic index, with hypercellularity and necrotic areas.

Diagnostic assumption: high grade spindle-cell sarcoma. Submitted to radiotherapy. The patient developed pain and a nodule in the right neck region. A new CT scan indicated lesion without vascular invasion and a nodule near the lung dome (probable recurrence). Submitted to palliative chemotherapy.

### Case 2

U.G.P., 39 years old, Caucasian, neck tumor for 6 months; we noticed a neck enlargement with bilateral masses that pushed the larynx to the right, almost extreme lateral. A patient with von Recklinghausen’s disease already submitted to resection of nodules in the chest and left shoulder. MRI showed a large solid neck tumor on the left side - compressing vessels, the larynx and trachea to the right side, all the way to the upper and anterior mediastinum, and large neck neuromas, larger on the left side. Nasal fibroscopy showed left vocal fold paralysis. The patient was taken to palliative surgery, in an attempt to decompress the trachea and avoid invasion of the large vessels. Macroscopically the lesion had 15.5 cm and weighed 350g. Histopathology: high grade spindle-cell sarcoma (malignant schwannoma). Immunohistochemistry test showed sarcoma.

## DISCUSSION

Pain and fast growth associated with neurofibromas leads us to suspect of sarcomatous transformation, although it can be a benign lesion^5^. In a study with 165 patients with malignant schwannomas (neurilemoma), no association was found between the disease and the use of tobaco^4^.

The cases reported are of patients with von Recklinghausen’s disease, aged between 37 and 40 years, males, non-smokers, with a fast growth lesion in the neck and supraclavicular regions.

Diagnostic investigation was carried out by image exams (CT, MRI and nasal fibroscopy) and histopathology.

Surgery was carried out, resecting the lesions and involved adjacent structures, added to adjuvant radio and/or chemotherapy.

Both cases were diagnosed and treated in a late stage, thus, unfortunately being of palliative nature only.

## FINAL REMARKS

These patients must be continuously followed up because of the possibility of the patient developing metastatic disease, especially in the lung, and have local recurrence.
